# Sex and gender reporting in RCTs of internet and mobile-based interventions for depression and anxiety in chronic conditions: A secondary analysis of a systematic review

**DOI:** 10.1371/journal.pmen.0000048

**Published:** 2024-07-25

**Authors:** Shaina Corrick, Emily Johnson, Serena Isley, Ben Vandermeer, Naomi Dolgoy, Jack Bates, Elana Godfrey, Cassidy Soltys, Conall Muir, Nicole Tegg, Colleen M. Norris, Puneeta Tandon

**Affiliations:** 1 Department of Medicine, Division of Gastroenterology (Liver Unit), University of Alberta, Edmonton, Alberta, Canada; 2 Department of Medicine, Epidemiology Coordinating and Research (EPICORE) Centre, University of Alberta, Edmonton, Alberta, Canada; 3 Faculty of Rehabilitation Science, University of Alberta, Edmonton, Alberta, Canada; 4 Faculty of Nursing, University of Alberta, Edmonton, Alberta, Canada; University for Continuing Education Krems: Universitat fur Weiterbildung Krems, AUSTRIA

## Abstract

Mind-body internet- and mobile-based intervention (IMIs) are gaining traction as scalable and effective strategies to manage mental health symptoms experienced by people living with chronic physical conditions. Sex and gender have implications for mind-body IMI participation, adherence, and efficacy. The objective of this secondary analysis was to assess the extent and nature of reporting of sex and/or gender in randomized controlled trials retrieved by a primary systematic review of mind-body IMIs assessing depression and anxiety symptoms among adults living with chronic physical conditions. The collected information included whether sex and gender-based analyses were carried out and explored the role of sex and gender on mental health outcomes, attrition, and recruitment rates. The protocol was registered with PROSPERO. A comprehensive search of six electronic databases was completed from database inception to March 2023. Sex and gender terms were summarized according to a standardized, three-point criteria: (1) non-binary use (i.e., > 2 categories used for both sex and gender definitions) (2) use of appropriate categories (i.e., sex = male/female/intersex, gender = man/woman/gender-diverse) and (3) non-interchangeable use of sex or gender terms throughout the citation. The use of sex and gender terms was deemed correct if all three criteria were met. The role of sex and gender on mental health outcomes, attrition and recruitment data were extracted where available. In the 56 included studies, 7691 participants were evaluated with a mean age of 43 years and 4780 (62%) were described as females/women. Two (4%) studies defined sex or gender using non-binary categorization. Twenty-eight (50%) studies used appropriate categories to define sex or gender. Twenty-five (45%) studies used sex and gender terms non-interchangeably. No studies met all three sex/gender criteria. Only one study provided stratified mental health scores by sex and/or gender within the publication. Eleven (20%) studies reported sex or gender imbalance as being a potential reason for outcome differences, with 3 studies conducting an adjusted statistical analysis investigating sex/gender as a moderator. Findings highlight low uptake of sex and gender considerations in the context of mind-body IMIs. Results underscore the need to incorporate guideline-based sex and gender terms and concepts, from data collection and analysis to reporting of evidence to inform mind-body IMI development and guide future research. Stratified sex and/or gender analyses are encouraged in future studies to assess intervention outcome differences.

## 1. Introduction

Globally, approximately 33% of adults live with at least one chronic physical condition (e.g., cardiac disease, chronic liver disease) [1]. Defined by the World Health Organization as *“conditions requiring ongoing management and treatment over extended periods of time*,” these conditions are closely linked to symptoms of depression and anxiety which in turn can decrease capacity for self-management [2, 3]. A 2020 qualitative systematic review of people living with chronic physical conditions highlighted that more steps must be taken to address unmet psychological and emotional needs of this patient population [4]. Sex and gender have garnered attention as critical factors in the experience of living with a chronic physical condition, and in the uptake and impact of clinical interventions [5, 6]. The Canadian Institute for Health Research (CIHR) defines sex as the classification of being biologically male, female, or intersex, including biological and/or physical characteristics such as reproductive anatomy and chromosomes [7]. The term gender is subject to change and exists on a continuum which includes identification as a man, woman or a gender-diverse identity. Gender also includes social constructs—such as identity, roles, relationships, and behaviours—and institutional-level factors—such as power relations and opportunities [7, 8].

Mental health conditions differ significantly across sex, with females developing depression, anxiety, and stress-related disorders more often than males [9, 10]. However, it is likely that this finding is complicated by gender-related factors such as stress, household responsibility, and societal pressures [11]. One qualitative study examined gender differences in the experiences of older adults with multimorbid conditions; despite gender similarities in mental health and quality of life, this study highlighted that men tended to discuss changes in personal power and control as a result of their chronic condition, whereas women reflected on the perceived impact of their chronic condition on their friends and family [12]. This highlights an important difference in how chronic conditions may be experienced differently due to gender roles and expectations. Emerging evidence also demonstrates worsened mental health outcomes among gender-diverse populations. Multiple studies have discussed the mental health burden that non-binary, transgender, and other gender-diverse populations may experience [13–15]. Gender-diverse populations may also experience higher rates of specific chronic conditions, such as depression, anxiety, and HIV compared to non-gender-diverse populations [16]. Overall, this presents a major point of discussion regarding health outcome research, as it highlights the importance of including expansive gender parameters and considering patient demographics beyond biological sex to fully examine the experience of individuals living with chronic conditions.

Mind-body interventions—which commonly include mindful movement (yoga, Tai-chi and qi gong), meditation, breathwork, and cognitive behavioural therapy (CBT)—have shown significant promise as non-pharmacological strategies to manage mental health symptoms and improve quality of life in individuals living with chronic physical conditions [17–20]. These interventions are amenable to digital delivery including by web and app-based platforms, herein referred to as internet- and mobile-based interventions (IMIs). While IMIs offer the convenience of increased access to content and services from home environments and across geographic boundaries, the use of IMIs differs across sex and gender, with women being more likely to participate compared to men [21]. This may be related in part to gender socialization for young women being towards interdependence, sensitivity and connectedness, and for young men being towards independence [22]. Participant sex has been identified as a significant predictor of IMI attrition rates, with males at a higher risk of attrition compared to females [21, 23]. Previous research also reports that men are less likely to access mental health services compared to women [24–26]. These sex and gender differences have been noted across pharmacotherapeutic and interventional research responses [27, 28].

Sex- and gender-based adjustments to IMI format and delivery has been associated with increased engagement and adherence [24, 25]. One qualitative study found that female participants strongly preferred a self-help delivery style, whereas male participants preferred to have information delivered in a video game format [29]. Consideration of sex and gender differences in the development and facilitation phase of mind-body IMIs can influence whether the intervention is suited for its intended audience [26]. In the analysis phase of research trials, the opportunity to account for sex, gender, and other demographics may offer important insight into acceptability, feasibility, adoption, sustainability, patient outcomes, and generalizability of results [30].

While previous systematic reviews have demonstrated the benefit of mind-body IMIs on mental health symptoms [20, 31–33], given the growing prevalence and potential of mind-body IMIs for widespread delivery, more information is needed about the influence of sex and gender. Current research has demonstrated that while gender-based considerations in research are becoming more prevalent, this area requires more attention. In a review of over 768 papers for the treatment of depression, less than 1% reported an a priori intention to analyze results by gender [34]. Gender and its associated factors have long been omitted as important factors of consideration regarding the outcomes of randomized controlled trials (RCTs). Within the context of a primary systematic review of RCTs that evaluated the impact of select mind-body IMIs on depression and anxiety symptoms in individuals living with chronic physical conditions [35], the objectives of this secondary analysis were to: 1) describe how sex and gender were reported across included studies and compare these descriptions according to guideline-based classification criteria [7, 36, 37]; 2) report on the frequency of the author stratification of anxiety and depression outcomes by sex and/or gender; and 3) provide a summary of sex and/or gender considerations related to participant recruitment and attrition rates.

## 2. Methods

### 2.1 Protocol

A separate protocol for this secondary analysis was not registered. Instead, the registration protocol for the primary systematic review [35] (PROSPERO #CRD42022375606) included secondary questions assessing sex and gender. Specifically, this included the proportion of studies reporting differences in mental health outcomes and intervention adherence rates across different sexes and/or genders, as well as accuracy and consistency of sex and gender reporting. This review follows the Preferred Reporting Items for Systematic Reviews and Meta-Analyses (PRISMA) guidelines [38].

### 2.2 Literature search

Electronic searches were conducted on October 4, 2022 across MEDLINE, EMBASE, SCOPUS, PsycInfo, CINAHL, and Cochrane Central Register of Controlled Trials (CENTRAL). Databases were searched from inception to present. Search results were updated on March 17, 2023. The complete search strategy for each database can be found in **S1 Appendix**. Keywords for this search include “mind-body therapies”, “cognitive behavioral therapy”, “wellness intervention”, “web based”, “remote”, “depression”, “anxiety” and “adult.” Records identified through database searches were exported in complete batches and duplicates were automatically removed upon import to the systematic review management software, Covidence [39]. In total 18,325 records were imported into Covidence from all databases and 7754 records were identified as duplicates, leaving 10,571 studies for title/abstract screening. In addition, hand searches were conducted among reference lists of relevant review articles [31, 40–42], though no studies were found for inclusion.

### 2.3 Eligibility criteria

The inclusion criteria of the primary systematic review has been published elsewhere and summarized in **Table 1** [35].

**Table 1:** Inclusion and exclusion criteria.

PICO-D Criteria	Inclusion Criteria	Exclusion Criteria
Population	• Adults (≥ 18 years) living with a chronic physical condition	• Adults with primarily chronic mental health conditions • Cancer survivors • Caregiver-only populations
Intervention	• Delivered through an internet or mobile-based platform • CBT-based interventions • Non-CBT interventions (e.g., breathwork, meditation, mindfulness, tai chi and/or yoga) • Combination CBT/non-CBT interventions	• DVD or teleconference delivery • Primarily in-person interventions
Control	• No intervention (treatment or care as usual) • Interventions not containing a mind-body technique	• Noninferiority trials
Outcomes	• Depression and/or anxiety outcomes utilizing psychometrically validated questionnaires	
Design	• Randomized controlled trials	• All other study designs

Only studies written in English were included. The search and study selection and inclusion process results are reported using a PRISMA flow diagram in the primary systematic review [35]. Title/abstract as well as full-text screening were performed by two independent reviewers, with disagreements resolved through a third-party reviewer.

### 2.4 Data extraction

#### 2.4.1 Sex and gender reporting

Data from the chosen articles were extracted independently using a form constructed in REDCap [43]. One author (SC) extracted data and another author (EJ) performed systematic verification checks. The primary objective was to summarize the reporting of sex and gender across studies and compare the reporting of sex and gender terms according to three-point evidence-based criteria previously published by Adisso et al. (2020), Gogovor et al. (2021), and developed with the CIHR framework and recommendations for defining sex and gender [7, 36, 37]. These guidelines take into account three criteria to assess accuracy of sex and gender reporting: (1) non-binary use (i.e., > 2 categories utilized for both sex and gender definitions) (2) use of appropriate categories (i.e., sex = male/female/intersex, gender = man/woman/gender-diverse/non-binary/gender-fluid) and (3) non-interchangeable use of sex or gender terms throughout the citation [36, 37]. The use of sex and gender terms was considered correct if all three criteria were met. All portions of the author’s manuscript were eligible for inclusion in this assessment and no adaptations were made to these criteria for the purpose of this analysis.

#### 2.4.2 Sex and gender stratified data: Enrollment, attrition, and mental health outcomes

Secondary objectives were to report on the frequency of stratification of anxiety and depression outcomes according to sex and gender and to describe sex or gender differences related to participant enrollment and attrition rates. The proportion of studies that provided sex and/or gender-stratified data on participant enrollment and attrition rates was identified using a binary coding scheme (yes/no). A narrative review of the results and discussion of each study was also conducted to synthesize any commentary around sex and/or gender differences in recruitment rates, attrition rates, or any perceived differences in outcomes according to sex or gender-related factors. For each study that did not include the mean change scores and associated standard deviation for depression and/or anxiety scores stratified by sex and/or gender (n = 55 studies), one attempt was made to contact authors by email in April 2023.

### 2.5 Risk of bias assessment

The risk of bias of each study was assessed using the revised Cochrane risk-of-bias tool for RCTs (version 2.0) [44]. Each of the five risk domains was scored against a three-point rating scale, corresponding to a low, moderate, and high risk of bias. Studies were included regardless of methodological quality.

### 2.6 Data analysis and synthesis

A descriptive analysis was conducted and reported using text, tables, and figures.

## 3. Results

### 3.1 Study inclusion

A total of 18,325 titles were retrieved through database searching. Following removal of duplicates, 10,571 titles were screened for eligibility. Of the remaining articles, 276 full-text articles were retrieved and assessed for eligibility. Two hundred and twenty were excluded, with reasons for exclusion being ineligible population (n = 54), ineligible intervention (n = 46), not original research (n = 39), ineligible control group (n = 37), ineligible outcome (n = 22), non-RCT study (n = 15), or missing data (n = 7). The primary systematic review includes the full PRISMA flow diagram [35]. The included 56 texts were critically appraised.

### 3.2 Methodological quality

Four (7%) studies were appraised to be at low risk of bias, 7 (13%) studies were found to be at high risk of bias. Most studies (45, 80%) were appraised to have at least one methodological concern. The majority studies had missing or incomplete outcome data due to participant drop out (44, 79%) (see **S2 Appendix**).

### 3.3 Characteristics of included studies

Fifty-six RCTs involving a total of 7691 study participants (mean = 137). Participants were described as female or women (n = 4780 (62%)), male or men (n = 2751 (36%)), or intersex or gender diverse (n = 2 (0.03%)). One feasibility study consisting of 158 participants did not provide a breakdown of participant sex or gender [45]. Most studies were conducted in the United States (10, 18%), Sweden (9, 16%), Netherlands (8, 14%) and the United Kingdom (9, 16%) with the remaining 20 trials conducted in other countries. Twenty-four different chronic physical conditions were identified; for the purposes of this analysis, these conditions were divided into four groupings: neurological conditions (17, 30%) (e.g., Parkinson’s disease) followed by cardiovascular conditions (e.g., heart failure) (7, 12%), cancer (6, 11%), and other (24, 43%) (e.g., HIV). Two (4%) trials enrolled participants living with multiple chronic conditions [46, 47] (**Table 2**). The most common mind-body IMIs were CBT (n = 28), followed by CBT with another mind-body technique (CBT+; n = 18), and non-CBT interventions (n = 10).

**Table 2:** Key study characteristics.

Author (Year)	Intervention Type	Country	Sample Size	Condition Type	% Female or Women	Sex/Gender Criterion Met
Ainsworth (2019) [48]	Non-CBT	United Kingdom	88	Other (Asthma)	60	2, 3
Ainsworth (2022) [45]	Non-CBT	United Kingdom	158	Other (Asthma)	Not available	None
Andersson (2002) [49]	CBT+	Sweden	117	Neurological	47	3
Bendig (2021) [50]	CBT	Germany	34	Cardiovascular	35	2, 3
Beukes (2018) [51]	CBT	United Kingdom	146	Neurological	43	None
Boeschoten (2017) [52]	CBT (Problem solving therapy)	Netherlands	171	Neurological	80	2, 3
Bromberg (2012) [53]	CBT+	United States of America	185	Neurological	89	None
Bundy (2013) [54]	CBT	United Kingdom	126	Other (Psoriasis)	53	2, 3
Burke (2019) [55]	CBT+	Ireland	69	Neurological	48	None
Cardol (2023) [56]	CBT	Netherlands	121	Other (Chronic kidney disease)	44	2, 3
Cooper (2011) [57]	CBT	United Kingdom	24	Neurological	75	None
Dear (2022) [46]	CBT	Australia	676	Multiple chronic conditions	89	1, 2
Devineni (2005) [58]	CBT+	United States of America	86	Neurological	89	None
Drozd (2014) [59]	Non-CBT	Norway	67	Other (HIV)	6	2
Ferwerda (2017) [60]	CBT	Netherlands	133	Other (Rheumatoid arthritis)	64	2, 3
Fischer (2015) [61]	CBT+	Germany	90	Neurological	78	None
Habibovic (2014) [62]	CBT	Netherlands	289	Cardiovascular	19	2
Hauffman (2020) [63]	CBT	Sweden	245	Cancer	71	2, 3
Hesser (2012) [64]	CBT	Sweden	99	Neurological	43	None
Hilmarsdottir (2020) [65]	Non-CBT	Iceland	30	Other (Diabetes)	37	2, 3
Huberty (2019) [66]	Non-CBT	United States of America	48	Cancer	93	None
Hunt (2009) [67]	CBT+	United States of America	121	Other (Irritable bowel syndrome)	81	2, 3
Hunt (2021) [68]	CBT	United States of America	54	Other (Irritable bowel syndrome)	75	None
Johansson (2019) [69]	CBT	Sweden	144	Cardiovascular	38	2, 3
Kraepelien (2020) [70]	CBT	Sweden	77	Neurological	61	2, 3
Kubo (2019) [71]	Non-CBT	United States of America	97	Cancer	68	2, 3
Kubo (2020) [72]	Non-CBT	United States of America	103	Cancer	70	None
Lee (2019) [73]	CBT+	Taiwan	160	Other (Irritable bowel syndrome)	100%	None
Ljotsson (2010) [74]	CBT+	Sweden	61	Other (Irritable bowel syndrome)	85	2, 3
Ljotsson (2011) [75]	CBT+	Sweden	86	Other (Irritable bowel syndrome)	74	3
Lundgren (2016) [76]	CBT	Sweden	50	Cardiovascular	42	2
McCombie (2016) [77]	CBT+	New Zealand	231	Other (Inflammatory bowel disease)	64	2, 3
Mensorio (2019) [78]	CBT	Spain	106	Other (Obesity)	44	3
Meyer (2019) [79]	CBT	Germany	200	Neurological	63	None
Migliorini (2016) [80]	CBT+	Australia	59	Neurological	29	None
Moss-Morris (2012) [81]	CBT	United Kingdom	45	Neurological	80	None
Motl (2017) [82]	CBT	United States of America	47	Neurological	85	2
Murray (2017) [83]	CBT+	United Kingdom	274	Other (Type II diabetes)	31	2
Nadort (2022) [84]	CBT	Netherlands	191	Other (Chronic kidney disease)	38	3
Neubert (2023) [85]	CBT+	Germany	172	Cancer	69	2
Newby (2017) [86]	CBT	Australia	106	Other (Type II diabetes)	71	None
Norlund (2018) [87]	CBT	Sweden	239	Cardiovascular	33	None
O’Moore (2018) [88]	CBT	Australia	77	Other (Knee osteoarthritis)	80	2, 3
Pagnini (2022) [89]	Non-CBT	United States of America	38	Neurological	50	3
Peerani (2022) [90]	CBT+	Canada	101	Other (Inflammatory bowel disease)	75	2, 3
Pottgen (2018) [91]	CBT+	Germany	275	Neurological	81	2, 3
Read (2020) [47]	CBT	Australia	308	Multiple chronic conditions	71	1, 3
Rini (2015) [92]	CBT	United States of America	113	Other (knee or hip osteoarthritis)	81	2
Rocamora-Gonzalez (2022) [93]	Non-CBT	Spain	102	Cancer	35	None
Schroder (2014) [94]	CBT+	Germany	78	Neurological	76	None
Schulz (2020) [95]	CBT	Germany	118	Cardiovascular	22	None
Van Beugen (2016) [96]	CBT	Netherlands	131	Other (Psoriasis)	49	None
Van Luenen (2018) [97]	CBT+	Netherlands	188	Other (HIV)	12	2, 3
Wadon (2021) [22]	CBT	United Kingdom	20	Neurological	75	2, 3
Young (2021) [98]	CBT+	Australia	125	Other (Obesity)	100	None
Younge (2015) [99]	Non-CBT	Netherlands	324	Cardiovascular	46	2

Abbreviations: Non-CBT = Non-Cognitive Behavioural Therapy; CBT = Cognitive Behavioural Therapy (CBT); CBT+ = Cognitive Behavioural Therapy + non-CBT technique

### 3.4 Sex and gender reporting

Two (4%) studies included a non-binary framework of assessing sex or gender. These studies were published in 2020, and 2022, respectively [46, 47] Twenty-eight (50%) studies categorized sex or gender using the appropriate category (sex = male/female/intersex, gender = man/woman/gender-diverse) [36, 37]. Twenty-five (45%) studies used sex and gender terms non-interchangeably. Studies fulfilling these two criteria ranged in publication year from 2009–2023. Twenty-one (38%) studies did not fulfill any of the sex or gender criteria. Of the studies that did exhibit sex or gender criteria, 13 (35%) fulfilled only one of the sex or gender criteria, and 21 (57%) fulfilled two. No studies fulfilled all three criteria. See **Table 3** for more information.

**Table 3:** Sex and gender reporting criteria and results.

Criteria	Met Criteria
Non-binary use (> 2 categories utilized for sex and gender)	2/56 (4%)
Use of appropriate categories (e.g., sex = male/female/intersex, gender = man/woman/gender-diverse)	28/56 (50%)
Non-interchangeable use of sex or gender terms throughout the citation	25/56 (45%)
Did not meet any of the above criteria	21/56 (38%)

### 3.5 Depression and anxiety outcomes

One study (2%) reported mean changes scores for anxiety stratified by sex or gender [92], with no authors reporting disaggregated sex/gender data for depression. As a result of email outreach to authors (n = 52), five authors responded with this data [46, 59, 61, 70, 92]; resulting in a total of four studies reporting sex/gender specific outcome data for depression, and three studies for anxiety. Two out of four studies reporting depression outcomes reported a greater decrease in mean scores for depression in females/women compared to males/men. For anxiety, all three studies reporting anxiety outcomes reported a greater decrease in mean change scores for females/women compared to males/men. Due to limited data availability, analysis of the stratified scores was not conducted. Of all studies included in the review, 11 (20%) studies discussed sex and/or gender as a potential reason for differences in anxiety and depression outcomes, the most common reason being attributed to differences in sex and/or gender ratios of participants within each study. Three studies included an adjusted analysis to investigate sex or gender as a moderator of anxiety or depression outcomes [53, 77, 87], with one out of three studies finding that sex was a significant moderator of treatment effects [77].

### 3.6 Sex and gender in recruitment and attrition rates

Six studies (11%) acknowledged that sex and/or gender imbalance within their participant pools served as a major limitation to their conclusions, with five of these studies citing greater females/women enrollment compared to males/men [53, 62, 70, 80, 90]. Kraepelien et al. further discussed this limitation of over-enrollment of females/women in mind-body IMIs within the context of disease incidence rates [70]. One (2%) study provided stratified sex or gender-based attrition data [92]. Authors of two studies (4%) also speculated on sex and gender differences regarding attrition rates [48, 79]. Authors reported discordant findings, with one reporting that females/women were more likely to drop out [48], and the other reporting that males/men were more likely to drop out [79]. Two studies discussed that gendered behaviours (e.g., willingness to reach out for support, reluctancy to engage in discussion around mental health) could impact participation and attrition rates [46, 80]. Dear et al. suggested that gender-based adjustment trends to chronic illness are generally unknown, however, it was possible that emotional distress levels may be higher among women with chronic health conditions [46]. Migliorini et al. discussed that even though men were overrepresented in spinal cord injury populations, their decreased participation in mind-body wellness interventions may be due to a gendered behavioral trend of reluctancy to seek support with emotional challenges [80].

## 4. Discussion

This secondary analysis assessed the reporting of sex and gender across 56 RCTs of mind-body IMIs on anxiety and depression symptoms in people living with a range of chronic physical conditions. Across studies, 4780 (62%) participants were described as female or women, 2751 (36%) were described as male or men, and 2 (0.03%) were described as intersex or gender diverse. None of the studies met all three criteria required to determine that sex and/or gender were accurately reported. Of the three criteria, half of the studies (n = 28, 50%) defined sex or gender using appropriate categories, less than half used sex and gender concepts non-interchangeably (n = 25, 45%). Very few (n = 2, 4%) defined sex or gender using a non-binary definition. Only one (2%) trial published sex or gender stratified data for anxiety outcomes within their report; no trials published sex or gender stratified data for depression outcomes, and 5 authors provided sex or gender-stratified data following an email request. Eleven studies described differences in recruitment or attrition trends by sex or gender within their reports, with five studies citing greater enrollment among females/women participants compared to male/men participants. Two studies that reported on sex and gender differences in attrition rates displayed disagreement, one suggesting that females/women were more likely to drop out and the other that males/men were more likely to drop out. These findings raise several points for discussion.

With 0% of studies using all three sex/gender criteria [36, 37], we have identified a low rate of detailed reporting on sex and gender within RCTs assessing anxiety and depression outcomes in participants exposed to mind-body IMIs. While our review represents the first evaluation of this data in mind-body IMIs, our findings are consistent with limited sex and/or gender reporting identified in several related reviews in the literature. A systematic evaluation of 133 Campbell reviews and 555 Cochrane reviews identified that less than 30% of series reported on sex and/or gender [100]. Others have come to similar conclusions regarding the low accuracy of sex and gender reporting, a meta-review assessing cardiovascular health interventions reporting that 0% of studies used sex and gender terms non-interchangeably [36, 101], and another systematic review of shared health care interventions for decision making reporting that 20% used these terms non-interchangeably [36].

Women and non-binary people, as well as females and intersex people have historically been neglected from health-related research. Repeated calls for greater attention to sex and gender within health-related research has made it imperative for studies to report their sex and gender demographics and analyses. When sex (male/female/intersex) and gender (man/woman/gender-diverse) are considered exclusively in binary terms (as occurred in 96% of our included studies), the generalizability of the study results are reduced to binary individuals and do not apply to individuals who are intersex or gender diverse [102]. This is of particular relevance with mental health initiatives as gender minorities face higher rates of mental health problems and unmet mental health needs compared to cisgender populations [13]. Unless focused recruitment strategies and data collection and reporting of diverse sexes and genders becomes a focus for research teams, it will remain a challenge to design effective mind-body IMIs that address these important drivers of mental distress [103–106].

Within our review, 45% of studies used sex and gender terms non-interchangeably. The high rates of interchangeable use (55%) and misclassification of sex and gender terms have important consequences, including confusion and inaccuracies in research and knowledge translation. Where sex is a biological difference, gender is a social construct. Reporting study results such as anxiety and depression symptoms which can so heavily be impacted by sex (e.g., hormones) [107] and gender (e.g., gender dysphoria, discrimination) risks inaccurately reporting the study results [108]. Additionally, when developing mind-body IMIs to manage anxiety and depression symptoms, accurately distinguishing between these two concepts is crucial for understanding health disparities and developing target mind-body IMIs [102]. Using these two terms interchangeably can lead to misleading or incomplete data and findings. Lastly, equating sex and gender can perpetuate societal norms and expectations related to gender roles and identities, hindering efforts to promote gender equality and inclusivity in health research and beyond.

Anxiety and depression data stratified by sex and/or gender was only reported in 1 (2%) study included in the current review, and for anxiety symptoms only. This presents a major limitation in determining how sex and gender may influence outcomes but is not uncommon. Consistent with our findings, in a 2017 review of Canadian RCTs involving human participants, only 6% of studies included any sex subgroup analysis, and only 4% reported sex-disaggregated data [30]. Without this data, we are unable to draw firm evidence-based conclusions about the sex and gender-specific impact of mind-body IMIs.

Despite the gaps in the previous literature, multiple measures are now being taken to improve sex and gender reporting in research. Targeted changes to common RCT practices can include more inclusivity of sex and gender considerations. First, the acknowledgement and distinction of sex and gender should be made within the RCT itself. Participants should be provided with multiple options to express both their biological sex as well as their gender. Current research is representing creative ways to capture participant gender quantitatively beyond gender identity, such as including targeted questions related to constructs such as gender roles, relations, and institutional factors that may influence study outcomes and experience [8]. Second, targeted recruitment of participants across genders is critical for promoting inclusivity within RCTs. Researchers hoping to champion gender diversity within their RCT sample can specify a targeted recruitment plan in their protocol, and pre-specify a post-hoc analysis of sex and gender [109]. Specific to examining mental health outcomes within RCTs, mixed methods approaches may also offer significant value in enhancing the assessment of participant gender and demographic factors on study outcomes [110]. Where possible, we recommend researchers incorporate both qualitative and quantitative measures to best incorporate the lived experiences and social determinants of participants within a sample. Some institutions such as the Canadian Institutes of Health Research have created measures to improve sex and gender-based outcomes by requiring research grant applicants to reflect on how they plan to include sex and gender within their proposed research [30, 111]. Other recommendations to increase sex and gender analysis within research include involving a “sex and gender champion” on each research team, which would either involve each team member taking a sex and gender in research course or having a team member become specialized in conducting sex and gender-based research [112]. This would ensure that trials have specialized methods and analysis protocols to improve the understanding of sex and gender-based differences. The consideration of sex as a biological variable is also a requirement in National Institutes of Health-funded research. Other initiatives have created standards for sex and gender reporting, including the criteria defined by Adisso et al. and Gogovor et al. [7, 36, 37] that were used in the current review, and the Sex and Gender Equity in Research (SAGER) guidelines [113]. While many journals have begun to include editorial policies for sex and gender analysis and guidelines such as SAGER, there are still opportunities to enhance and improve reporting policies [28, 113, 114]. A 2021 review of leading general medical and global health journals found that 5 out of 14 journals required a specific sex and gender analysis within the research, with the inclusion of sex and gender typically not extending beyond reporting the basic demographics of participants [115].

With nearly 2/3 of the participants in this secondary analysis reported as being female or women, the results highlight that recruitment of other sexes and genders living with chronic physical conditions into mind-body IMIs can be unequal. By accounting for the unique barriers that participants of different sexes and genders may experience in accessing mind-body IMIs, improved recruitment, attrition rates, and outcomes may be seen. For example, Young et al. developed a gender-based online eHealth intervention (“SHED-IT”) specifically for men living with obesity, recognizing the unique barriers that men may face in this space, such as gender-specific stigma around mental health and lower mental health access rates [98]. This intervention was tailored to men at both the surface level (e.g., representation of men participating in wellness activities) and also at the core of the intervention, intentionally using more direct communication styles combined with light-hearted humor, which was intended to enhance program experiences based on evidence-based communication styles that men may respond more preferably towards [98, 116]. The program was found to be effective for participant depression and anxiety outcomes [98]. Lee et al. used a sex and gender-based approach when designing a mind-body IMI (“Mind Over Mood”) for female irritable bowel syndrome (IBS) patients [73]. This approach was taken with consideration of the 2:1 female-to-male incidence ratio for IBS and the recognition of greater levels of emotional distress present in female patients, which may contribute to the development and progression of IBS. Lee et al. tailored the intervention by incorporating mind-body practices that have been documented to be helpful in relieving emotional distress, such as expressive writing, and found statistically significant improvements in depression and anxiety outcomes [73]. Consequently, there may be significant value in tailoring interventions by sex and gender in terms of level of patient acceptability as well as mental health outcomes.

## 5. Limitations

This secondary analysis should be interpreted in the context of the underlying evidence base and analysis limitations. First, in the context of the evidence base, some studies were of low to moderate quality [44], and most required outreach to authors due to the limited availability of complete data. This may have affected the analysis by introducing sampling bias. Second, most evidence was extracted from studies conducted in higher-income countries, limiting the reflection of global heterogeneity in sex and gender definitions. Due to resource limitations for translation, analysis was limited to studies published in English. Third, the definition of gender used in this secondary analysis was largely limited to capturing the construct of gender identity; gender also includes constructs such as gender roles, relationships, and institution-level factors [8]. This data was not collected due to its limited availability in the included studies, but it should be considered in future studies. Fourth, in the broader published literature, there has been a steady rise of sex and gender-based analyses [117] and subsequent definitions for sex and gender [118]. It is recognized that definitions of sex and gender diversity may not have been as available (e.g., an option on a demographic survey) or deemed as important in clinical trials as it is in the present day [119]. As a result, while this secondary analysis can be used as a lens to interpret previous studies, it may more importantly be seen as a reference point for clinicians looking to conduct future mind-body IMI research amongst people living with chronic physical conditions.

## 6. Conclusion

With increasing access to internet and mobile tools, mind-body IMIs are anticipated to continue to rise in prevalence. In this context, it is essential that we equitably service the needs of the individuals using these interventions. This secondary analysis of a systematic review and meta-analysis of RCTs investigating mind-body IMIs reveals that no studies satisfied all three sex and gender-based reporting criteria, with 4% providing >2 categories for sex and gender, 45% using sex and gender categories non-interchangeably, and 50% using appropriate categories for sex and gender. To further our understanding of the impact of mind-body IMIs on symptoms of anxiety and depression, the interplay of biological sex, psychological, and social domains in the context of gender need to be prioritized both in intervention design and analysis. A sex and gender-sensitive approach holds promise to reduce health inequities, gain a better understanding of the effect of mind-body IMIs on anxiety and depression symptoms, identify the characteristics of individuals who are likely to get the most benefit, and design interventions that are inclusive for the needs of diverse sexes and/or genders. Future studies investigating mind-body IMIs can use the findings of this secondary analysis to assist in intervention design and protocol development.

## Supporting information

S1 Appendix Full search strategy. (DOCX)

S2 Appendix Risk of bias assessment. (DOCX)

S1 Data Risk of bias data. (XLSX)

## References

[1] Hajat C, Stein E. The global burden of multiple chronic conditions: A narrative review. Prev Med Rep. 2018;12:284–93. doi: 10.1016/j.pmedr.2018.10.008 30406006 PMC6214883

[2] Chapman DP, Perry GS, Strine TW. The vital link between chronic disease and depressive disorders. Prev Chronic Dis. 2005;2(1):A14. 15670467 PMC1323317

[3] Lin EH, Katon W, Von Korff M, Rutter C, Simon GE, Oliver M, et al. Relationship of depression and diabetes self-care, medication adherence, and preventive care. Diabetes Care. 2004;27(9):2154–60. doi: 10.2337/diacare.27.9.2154 15333477

[4] Eassey D, Reddel HK, Ryan K, Smith L. ’It is like learning how to live all over again’ A systematic review of people’s experiences of living with a chronic illness from a self-determination theory perspective. Health Psychol Behav Med. 2020;8(1):270–91. doi: 10.1080/21642850.2020.1794879 34040872 PMC8143269

[5] Mauvais-Jarvis F, Bairey Merz N, Barnes PJ, Brinton RD, Carrero JJ, DeMeo DL, et al. Sex and gender: modifiers of health, disease, and medicine. Lancet. 2020;396(10250):565–82. doi: 10.1016/S0140-6736(20)31561-0 32828189 PMC7440877

[6] van Diemen J, Verdonk P, Chieffo A, Regar E, Mauri F, Kunadian V, et al. The importance of achieving sex- and gender-based equity in clinical trials: a call to action. Eur Heart J. 2021;42(31):2990–4. doi: 10.1093/eurheartj/ehab457 34352884 PMC8370758

[7] Research CIoH. What is gender? What is sex? 2023.

[8] Tadiri CP, Raparelli V, Abrahamowicz M, Kautzy-Willer A, Kublickiene K, Herrero MT, et al. Methods for prospectively incorporating gender into health sciences research. J Clin Epidemiol. 2021;129:191–7. doi: 10.1016/j.jclinepi.2020.08.018 32980428

[9] Kuehner C. Why is depression more common among women than among men? Lancet Psychiatry. 2017;4(2):146–58. doi: 10.1016/S2215-0366(16)30263-2 27856392

[10] Li SH, Graham BM. Why are women so vulnerable to anxiety, trauma-related and stress-related disorders? The potential role of sex hormones. Lancet Psychiatry. 2017;4(1):73–82. doi: 10.1016/S2215-0366(16)30358-3 27856395

[11] Bird CE, Rieker PP. Gender matters: an integrated model for understanding men’s and women’s health. Soc Sci Med. 1999;48(6):745–55. doi: 10.1016/s0277-9536(98)00402-x 10190637

[12] Clarke LH, Bennett E. ‘You learn to live with all the things that are wrong with you’: gender and the experience of multiple chronic conditions in later life. Ageing and Society. 2013;33(2):342–60. doi: 10.1017/S0144686X11001243 24976658 PMC4072648

[13] Stanton AM, Batchelder AW, Kirakosian N, Scholl J, King D, Grasso C, et al. Differences in mental health symptom severity and care engagement among transgender and gender diverse individuals: Findings from a large community health center. PLoS One. 2021;16(1):e0245872. doi: 10.1371/journal.pone.0245872 33493207 PMC7833136

[14] Canvin L, Twist J, Solomons W. How do mental health professionals describe their experiences of providing care for gender diverse adults? A systematic literature review. Psychology & Sexuality. 2022;13(3):717–41.

[15] Watkinson RE, Linfield A, Tielemans J, Francetic I, Munford L. Gender-related self-reported mental health inequalities in primary care in England: a cross-sectional analysis using the GP Patient Survey. The Lancet Public Health. 2024;9(2):e100–e8. doi: 10.1016/S2468-2667(23)00301-8 38307677

[16] Tran NK, Lunn MR, Schulkey CE, Tesfaye S, Nambiar S, Chatterjee S, et al. Prevalence of 12 Common Health Conditions in Sexual and Gender Minority Participants in the All of Us Research Program. JAMA Netw Open. 2023;6(7):e2324969. doi: 10.1001/jamanetworkopen.2023.24969 37523187 PMC10391317

[17] Oyedeji AD, Ullah I, Weich S, Bentall R, Booth A. Effectiveness of non-specialist delivered psychological interventions on glycemic control and mental health problems in individuals with type 2 diabetes: a systematic review and meta-analysis. Int J Ment Health Syst. 2022;16(1):9. doi: 10.1186/s13033-022-00521-2 35120528 PMC8817494

[18] White V, Linardon J, Stone JE, Holmes-Truscott E, Olive L, Mikocka-Walus A, et al. Online psychological interventions to reduce symptoms of depression, anxiety, and general distress in those with chronic health conditions: a systematic review and meta-analysis of randomized controlled trials. Psychological Medicine. 2022;52(3):548–73. doi: 10.1017/S0033291720002251 32674747

[19] Sanogo F, Xu K, Cortessis VK, Weigensberg MJ, Watanabe RM. Mind- and Body-Based Interventions Improve Glycemic Control in Patients with Type 2 Diabetes: A Systematic Review and Meta-Analysis. Journal of Integrative and Complementary Medicine. 2022;29(2):69–79. doi: 10.1089/jicm.2022.0586 36070591 PMC13134557

[20] Liu Z, Jia Y, Li M, Meng X, Shang B, Wang C, et al. Effectiveness of online mindfulness-based interventions for improving mental health in patients with physical health conditions: Systematic review and meta-analysis. Arch Psychiatr Nurs. 2022;37:52–60. doi: 10.1016/j.apnu.2021.10.001 35337439

[21] Karyotaki E, Kleiboer A, Smit F, Turner DT, Pastor AM, Andersson G, et al. Predictors of treatment dropout in self-guided web-based interventions for depression: an ’individual patient data’ meta-analysis. Psychol Med. 2015;45(13):2717–26. doi: 10.1017/S0033291715000665 25881626

[22] Wadon ME, MacIver C, Winter M, Peall KJ. Internet-based cognitive behavioural therapy as a feasible treatment of adult-onset, focal, isolated, idiopathic cervical dystonia. Clin Park Relat Disord. 2021;5:100121. doi: 10.1016/j.prdoa.2021.100121 34927048 PMC8649077

[23] Schmidt ID, Forand NR, Strunk DR. Predictors of Dropout in Internet-Based Cognitive Behavioral Therapy for Depression. Cognit Ther Res. 2019;43(3):620–30. doi: 10.1007/s10608-018-9979-5 32879540 PMC7462416

[24] Mackenzie CS, Gekoski WL, Knox VJ. Age, gender, and the underutilization of mental health services: the influence of help-seeking attitudes. Aging Ment Health. 2006;10(6):574–82. doi: 10.1080/13607860600641200 17050086

[25] Sagar-Ouriaghli I, Godfrey E, Bridge L, Meade L, Brown JSL. Improving Mental Health Service Utilization Among Men: A Systematic Review and Synthesis of Behavior Change Techniques Within Interventions Targeting Help-Seeking. Am J Mens Health. 2019;13(3):1557988319857009. doi: 10.1177/1557988319857009 31184251 PMC6560805

[26] Runnels V, Tudiver S, Doull M, Boscoe M. The challenges of including sex/gender analysis in systematic reviews: a qualitative survey. Syst Rev. 2014;3:33. doi: 10.1186/2046-4053-3-33 24720875 PMC3990268

[27] Day S, Mason R, Lagosky S, Rochon PA. Integrating and evaluating sex and gender in health research. Health Res Policy Syst. 2016;14(1):75. doi: 10.1186/s12961-016-0147-7 27724961 PMC5057373

[28] De Castro P, Heidari S, Babor TF. Sex And Gender Equity in Research (SAGER): reporting guidelines as a framework of innovation for an equitable approach to gender medicine. Commentary. Ann Ist Super Sanita. 2016;52(2):154–7. doi: 10.4415/ANN_16_02_05 27364388

[29] Smail-Crevier R, Powers G, Noel C, Wang J. Health-Related Internet Usage and Design Feature Preference for E-Mental Health Programs Among Men and Women. J Med Internet Res. 2019;21(3):e11224. doi: 10.2196/11224 30882361 PMC6441854

[30] Welch V, Doull M, Yoganathan M, Jull J, Boscoe M, Coen SE, et al. Reporting of sex and gender in randomized controlled trials in Canada: a cross-sectional methods study. Res Integr Peer Rev. 2017;2:15. doi: 10.1186/s41073-017-0039-6 29451565 PMC5803639

[31] Toivonen KI, Zernicke K, Carlson LE. Web-Based Mindfulness Interventions for People With Physical Health Conditions: Systematic Review. J Med Internet Res. 2017;19(8):e303. doi: 10.2196/jmir.7487 28860106 PMC5599726

[32] Zhang A, Jing S, Solomon P, Brose A. Mindfulness-Based Intervention for Chinese Breast Cancer Patients: A Systematic Review and Meta-Analysis. Research on Social Work Practice. 2021;31(7):683–92.

[33] Montañés-Masias B, Bort-Roig J, Pascual JC, Soler J, Briones-Buixassa L. Online psychological interventions to improve symptoms in multiple sclerosis: A systematic review: Online psychological interventions in Multiple Sclerosis. Acta Neurol Scand. 2022;146(5):448–64. doi: 10.1111/ane.13709 36121184 PMC9825977

[34] Weinberger AH, McKee SA, Mazure CM. Inclusion of women and gender-specific analyses in randomized clinical trials of treatments for depression. J Womens Health (Larchmt). 2010;19(9):1727–32. doi: 10.1089/jwh.2009.1784 20799923 PMC2936499

[35] Johnson E, Corrick S, Isley S, Vandermeer B, Dolgoy N, Bates J, et al. Mind-body internet and mobile-based interventions for depression and anxiety in adults with chronic physical conditions: A systematic review of RCTs. PLOS Digital Health. 2024;3(1):e0000435. doi: 10.1371/journal.pdig.0000435 38261600 PMC10805319

[36] Adisso É L, Zomahoun HTV, Gogovor A, Légaré F. Sex and gender considerations in implementation interventions to promote shared decision making: A secondary analysis of a Cochrane systematic review. PLoS One. 2020;15(10):e0240371. doi: 10.1371/journal.pone.0240371 33031475 PMC7544054

[37] Gogovor A, Zomahoun HTV, Ekanmian G, Adisso É L, Deom Tardif A, Khadhraoui L, et al. Sex and gender considerations in reporting guidelines for health research: a systematic review. Biol Sex Differ. 2021;12(1):62. doi: 10.1186/s13293-021-00404-0 34801060 PMC8605583

[38] Page MJ, McKenzie JE, Bossuyt PM, Boutron I, Hoffmann TC, Mulrow CD, et al. The PRISMA 2020 statement: an updated guideline for reporting systematic reviews. BMJ. 2021;372:n71. doi: 10.1136/bmj.n71 33782057 PMC8005924

[39] Innovation VH, inventorCovidence Systematic Review Software. Melbourne, Australia.

[40] Akyirem S, Forbes A, Wad JL, Due-Christensen M. Psychosocial interventions for adults with newly diagnosed chronic disease: A systematic review. Journal of Health Psychology. 2021;27(7):1753–82. doi: 10.1177/1359105321995916 33586486 PMC9092922

[41] Sasseville M, LeBlanc A, Boucher M, Dugas M, Mbemba G, Tchuente J, et al. Digital health interventions for the management of mental health in people with chronic diseases: a rapid review. BMJ Open. 2021;11(4):e044437. doi: 10.1136/bmjopen-2020-044437 33820786 PMC8030477

[42] Beatty L, Lambert S. A systematic review of internet-based self-help therapeutic interventions to improve distress and disease-control among adults with chronic health conditions. Clin Psychol Rev. 2013;33(4):609–22. doi: 10.1016/j.cpr.2013.03.004 23603521

[43] Harris PA, Taylor R, Minor BL, Elliott V, Fernandez M, O’Neal L, et al. The REDCap consortium: Building an international community of software platform partners. J Biomed Inform. 2019;95:103208. doi: 10.1016/j.jbi.2019.103208 31078660 PMC7254481

[44] Higgins JP, Altman DG, Gøtzsche PC, Jüni P, Moher D, Oxman AD, et al. The Cochrane Collaboration’s tool for assessing risk of bias in randomised trials. Bmj. 2011;343:d5928. doi: 10.1136/bmj.d5928 22008217 PMC3196245

[45] Ainsworth B, Stanescu S, Stuart B, Russell D, Liddiard M, Djukanovic R, et al. A feasibility trial of a digital mindfulness-based intervention to improve asthma-related quality of life for primary care patients with asthma. J Behav Med. 2022;45(1):133–47. doi: 10.1007/s10865-021-00249-3 34448986 PMC8818629

[46] Dear BF, Scott AJ, Fogliati R, Gandy M, Karin E, Dudeney J, et al. The Chronic Conditions Course: A Randomised Controlled Trial of an Internet-Delivered Transdiagnostic Psychological Intervention for People with Chronic Health Conditions. Psychother Psychosom. 2022;91(4):265–76. doi: 10.1159/000522530 35367986

[47] Read J, Sharpe L, Burton AL, Arean PA, Raue PJ, McDonald S, et al. A randomized controlled trial of internet-delivered cognitive behaviour therapy to prevent the development of depressive disorders in older adults with multimorbidity. J Affect Disord. 2020;264:464–73. doi: 10.1016/j.jad.2019.11.077 31767215

[48] Ainsworth B, Greenwell K, Stuart B, Raftery J, Mair F, Bruton A, et al. Feasibility trial of a digital self-management intervention ’My Breathing Matters’ to improve asthma-related quality of life for UK primary care patients with asthma. BMJ Open. 2019;9(11):e032465. doi: 10.1136/bmjopen-2019-032465 31722952 PMC6858238

[49] Andersson G, Strömgren T, Ström L, Lyttkens L. Randomized controlled trial of internet-based cognitive behavior therapy for distress associated with tinnitus. Psychosom Med. 2002;64(5):810–6. doi: 10.1097/01.psy.0000031577.42041.f8 12271112

[50] Bendig E, Bauereiß N, Buntrock C, Habibović M, Ebert DD, Baumeister H. Lessons learned from an attempted randomized-controlled feasibility trial on "WIDeCAD"—An internet-based depression treatment for people living with coronary artery disease (CAD). Internet Interv. 2021;24:100375. doi: 10.1016/j.invent.2021.100375 33732627 PMC7941156

[51] Beukes EW, Baguley DM, Allen PM, Manchaiah V, Andersson G. Audiologist-Guided Internet-Based Cognitive Behavior Therapy for Adults With Tinnitus in the United Kingdom: A Randomized Controlled Trial. Ear Hear. 2018;39(3):423–33. doi: 10.1097/AUD.0000000000000505 29095725

[52] Boeschoten RE, Dekker J, Uitdehaag BM, Beekman AT, Hoogendoorn AW, Collette EH, et al. Internet-based treatment for depression in multiple sclerosis: A randomized controlled trial. Mult Scler. 2017;23(8):1112–22. doi: 10.1177/1352458516671820 27682229

[53] Bromberg J, Wood ME, Black RA, Surette DA, Zacharoff KL, Chiauzzi EJ. A randomized trial of a web-based intervention to improve migraine self-management and coping. Headache. 2012;52(2):244–61. doi: 10.1111/j.1526-4610.2011.02031.x 22413151 PMC3305283

[54] Bundy C, Pinder B, Bucci S, Reeves D, Griffiths CE, Tarrier N. A novel, web-based, psychological intervention for people with psoriasis: the electronic Targeted Intervention for Psoriasis (eTIPs) study. Br J Dermatol. 2013;169(2):329–36. doi: 10.1111/bjd.12350 23551271

[55] Burke D, Lennon O, Blake C, Nolan M, Barry S, Smith E, et al. An internet-delivered cognitive behavioural therapy pain management programme for spinal cord injury pain: A randomized controlled trial. Eur J Pain. 2019;23(7):1264–82. doi: 10.1002/ejp.1402 31002442

[56] Cardol CK, van Middendorp H, Dusseldorp E, van der Boog PJM, Hilbrands LB, Navis G, et al. eHealth to Improve Psychological Functioning and Self-Management of People With Chronic Kidney Disease: A Randomized Controlled Trial. Psychosom Med. 2023;85(2):203–15. doi: 10.1097/PSY.0000000000001163 36662615 PMC9924966

[57] Cooper CL, Hind D, Parry GD, Isaac CL, Dimairo M, O’Cathain A, et al. Computerised cognitive behavioural therapy for the treatment of depression in people with multiple sclerosis: external pilot trial. Trials. 2011;12:259. doi: 10.1186/1745-6215-12-259 22168507 PMC3272061

[58] Devineni T, Blanchard EB. A randomized controlled trial of an internet-based treatment for chronic headache. Behav Res Ther. 2005;43(3):277–92. doi: 10.1016/j.brat.2004.01.008 15680926

[59] Drozd F, Skeie LG, Kraft P, Kvale D. A web-based intervention trial for depressive symptoms and subjective well-being in patients with chronic HIV infection. AIDS Care. 2014;26(9):1080–9. doi: 10.1080/09540121.2013.869541 24359563

[60] Ferwerda M, van Beugen S, van Middendorp H, Spillekom-van Koulil S, Donders ART, Visser H, et al. A tailored-guided internet-based cognitive-behavioral intervention for patients with rheumatoid arthritis as an adjunct to standard rheumatological care: results of a randomized controlled trial. Pain. 2017;158(5):868–78. doi: 10.1097/j.pain.0000000000000845 28106666

[61] Fischer A, Schröder J, Vettorazzi E, Wolf OT, Pöttgen J, Lau S, et al. An online programme to reduce depression in patients with multiple sclerosis: a randomised controlled trial. Lancet Psychiatry. 2015;2(3):217–23. doi: 10.1016/S2215-0366(14)00049-2 26359900

[62] Habibović M, Denollet J, Cuijpers P, Spek VR, van den Broek KC, Warmerdam L, et al. E-health to manage distress in patients with an implantable cardioverter-defibrillator: primary results of the WEBCARE trial. Psychosom Med. 2014;76(8):593–602. doi: 10.1097/PSY.0000000000000096 25264974

[63] Hauffman A, Alfonsson S, Bill-Axelson A, Bergkvist L, Forslund M, Mattsson S, et al. Cocreated internet-based stepped care for individuals with cancer and concurrent symptoms of anxiety and depression: Results from the U-CARE AdultCan randomized controlled trial. Psychooncology. 2020;29(12):2012–8. doi: 10.1002/pon.5489 32691455 PMC7821133

[64] Hesser H, Gustafsson T, Lundén C, Henrikson O, Fattahi K, Johnsson E, et al. A randomized controlled trial of Internet-delivered cognitive behavior therapy and acceptance and commitment therapy in the treatment of tinnitus. J Consult Clin Psychol. 2012;80(4):649–61. doi: 10.1037/a0027021 22250855

[65] Hilmarsdóttir E, Sigurðardóttir Á K, Arnardóttir RH. A Digital Lifestyle Program in Outpatient Treatment of Type 2 Diabetes: A Randomized Controlled Study. J Diabetes Sci Technol. 2021;15(5):1134–41. doi: 10.1177/1932296820942286 32680441 PMC8442170

[66] Huberty J, Eckert R, Dueck A, Kosiorek H, Larkey L, Gowin K, et al. Online yoga in myeloproliferative neoplasm patients: results of a randomized pilot trial to inform future research. BMC Complement Altern Med. 2019;19(1):121. doi: 10.1186/s12906-019-2530-8 31174535 PMC6556039

[67] Hunt MG, Moshier S, Milonova M. Brief cognitive-behavioral internet therapy for irritable bowel syndrome. Behav Res Ther. 2009;47(9):797–802. doi: 10.1016/j.brat.2009.05.002 19570525

[68] Hunt M, Miguez S, Dukas B, Onwude O, White S. Efficacy of Zemedy, a Mobile Digital Therapeutic for the Self-management of Irritable Bowel Syndrome: Crossover Randomized Controlled Trial. JMIR Mhealth Uhealth. 2021;9(5):e26152. doi: 10.2196/26152 33872182 PMC8176342

[69] Johansson P, Westas M, Andersson G, Alehagen U, Broström A, Jaarsma T, et al. An Internet-Based Cognitive Behavioral Therapy Program Adapted to Patients With Cardiovascular Disease and Depression: Randomized Controlled Trial. JMIR Ment Health. 2019;6(10):e14648. doi: 10.2196/14648 31584000 PMC7020777

[70] Kraepelien M, Schibbye R, Månsson K, Sundström C, Riggare S, Andersson G, et al. Individually Tailored Internet-Based Cognitive-Behavioral Therapy for Daily Functioning in Patients with Parkinson’s Disease: A Randomized Controlled Trial. J Parkinsons Dis. 2020;10(2):653–64. doi: 10.3233/JPD-191894 32176657 PMC7242852

[71] Kubo A, Kurtovich E, McGinnis M, Aghaee S, Altschuler A, Quesenberry C Jr., et al. A Randomized Controlled Trial of mHealth Mindfulness Intervention for Cancer Patients and Informal Cancer Caregivers: A Feasibility Study Within an Integrated Health Care Delivery System. Integr Cancer Ther. 2019;18:1534735419850634. doi: 10.1177/1534735419850634 31092044 PMC6537293

[72] Kubo A, Kurtovich E, McGinnis M, Aghaee S, Altschuler A, Quesenberry C, Jr., et al. Pilot pragmatic randomized trial of mHealth mindfulness-based intervention for advanced cancer patients and their informal caregivers. Psychooncology. 2020. doi: 10.1002/pon.5557 32979294

[73] Lee TY, Hsieh TC, Sung HC, Chen WL. Internet-Delivered Cognitive Behavior Therapy for Young Taiwanese Female Nursing Students with Irritable Bowel Syndrome-A Cluster Randomized Controlled Trial. Int J Environ Res Public Health. 2019;16(5). doi: 10.3390/ijerph16050708 30818837 PMC6427663

[74] Ljótsson B, Falk L, Vesterlund AW, Hedman E, Lindfors P, Rück C, et al. Internet-delivered exposure and mindfulness based therapy for irritable bowel syndrome—a randomized controlled trial. Behav Res Ther. 2010;48(6):531–9. doi: 10.1016/j.brat.2010.03.003 20362976

[75] Ljótsson B, Andersson G, Andersson E, Hedman E, Lindfors P, Andréewitch S, et al. Acceptability, effectiveness, and cost-effectiveness of internet-based exposure treatment for irritable bowel syndrome in a clinical sample: a randomized controlled trial. BMC Gastroenterol. 2011;11:110. doi: 10.1186/1471-230X-11-110 21992655 PMC3206465

[76] Lundgren JG, Dahlström Ö, Andersson G, Jaarsma T, Kärner Köhler A, Johansson P. The Effect of Guided Web-Based Cognitive Behavioral Therapy on Patients With Depressive Symptoms and Heart Failure: A Pilot Randomized Controlled Trial. J Med Internet Res. 2016;18(8):e194. doi: 10.2196/jmir.5556 27489077 PMC5070581

[77] McCombie A, Gearry R, Andrews J, Mulder R, Mikocka-Walus A. Does Computerized Cognitive Behavioral Therapy Help People with Inflammatory Bowel Disease? A Randomized Controlled Trial. Inflamm Bowel Dis. 2016;22(1):171–81. doi: 10.1097/MIB.0000000000000567 26360545

[78] Mensorio MS, Cebolla-Martí A, Rodilla E, Palomar G, Lisón JF, Botella C, et al. Analysis of the efficacy of an internet-based self-administered intervention ("Living Better") to promote healthy habits in a population with obesity and hypertension: An exploratory randomized controlled trial. Int J Med Inform. 2019;124:13–23. doi: 10.1016/j.ijmedinf.2018.12.007 30784422

[79] Meyer B, Weiss M, Holtkamp M, Arnold S, Brückner K, Schröder J, et al. Effects of an epilepsy-specific Internet intervention (Emyna) on depression: Results of the ENCODE randomized controlled trial. Epilepsia. 2019;60(4):656–68. doi: 10.1111/epi.14673 30802941

[80] Migliorini C, Sinclair A, Brown D, Tonge B, New P. A randomised control trial of an Internet-based cognitive behaviour treatment for mood disorder in adults with chronic spinal cord injury. Spinal Cord. 2016;54(9):695–701. doi: 10.1038/sc.2015.221 26690861

[81] Moss-Morris R, McCrone P, Yardley L, van Kessel K, Wills G, Dennison L. A pilot randomised controlled trial of an Internet-based cognitive behavioural therapy self-management programme (MS Invigor8) for multiple sclerosis fatigue. Behav Res Ther. 2012;50(6):415–21. doi: 10.1016/j.brat.2012.03.001 22516321

[82] Motl RW, Hubbard EA, Bollaert RE, Adamson BC, Kinnett-Hopkins D, Balto JM, et al. Randomized controlled trial of an e-learning designed behavioral intervention for increasing physical activity behavior in multiple sclerosis. Mult Scler J Exp Transl Clin. 2017;3(4):2055217317734886. doi: 10.1177/2055217317734886 29051831 PMC5637983

[83] Murray E, Sweeting M, Dack C, Pal K, Modrow K, Hudda M, et al. Web-based self-management support for people with type 2 diabetes (HeLP-Diabetes): randomised controlled trial in English primary care. BMJ Open. 2017;7(9):e016009. doi: 10.1136/bmjopen-2017-016009 28954789 PMC5623569

[84] Nadort E, Schouten RW, Boeschoten RE, Smets Y, Chandie Shaw P, Vleming LJ, et al. Internet-based treatment for depressive symptoms in hemodialysis patients: A cluster randomized controlled trial. Gen Hosp Psychiatry. 2022;75:46–53. doi: 10.1016/j.genhosppsych.2022.01.008 35134703

[85] Neubert S, Schlecht S, Meng K, Rabe A, Jentschke E. Effects of a Video Sequence Based Intervention on Anxiety, Fatigue and Depression in Cancer Patients: Results of a Randomized Controlled Trial. Integr Cancer Ther. 2023;22:15347354231153172. doi: 10.1177/15347354231153172 36799503 PMC9940180

[86] Newby J, Robins L, Wilhelm K, Smith J, Fletcher T, Gillis I, et al. Web-Based Cognitive Behavior Therapy for Depression in People With Diabetes Mellitus: A Randomized Controlled Trial. J Med Internet Res. 2017;19(5):e157. doi: 10.2196/jmir.7274 28506956 PMC5447827

[87] Norlund F, Wallin E, Olsson EMG, Wallert J, Burell G, von Essen L, et al. Internet-Based Cognitive Behavioral Therapy for Symptoms of Depression and Anxiety Among Patients With a Recent Myocardial Infarction: The U-CARE Heart Randomized Controlled Trial. J Med Internet Res. 2018;20(3):e88. doi: 10.2196/jmir.9710 29519777 PMC5874001

[88] O’Moore K A, Newby JM, Andrews G, Hunter DJ, Bennell K, Smith J, et al. Internet Cognitive-Behavioral Therapy for Depression in Older Adults With Knee Osteoarthritis: A Randomized Controlled Trial. Arthritis Care Res (Hoboken). 2018;70(1):61–70. doi: 10.1002/acr.23257 28426917

[89] Pagnini F, Phillips D, Haulman A, Bankert M, Simmons Z, Langer E. An online non-meditative mindfulness intervention for people with ALS and their caregivers: a randomized controlled trial. Amyotroph Lateral Scler Frontotemporal Degener. 2022;23(1–2):116–27. doi: 10.1080/21678421.2021.1928707 34027769

[90] Peerani F, Watt M, Ismond KP, Whitlock R, Ambrosio L, Hotte N, et al. A randomized controlled trial of a multicomponent online stress reduction intervention in inflammatory bowel disease. Therap Adv Gastroenterol. 2022;15:17562848221127238. doi: 10.1177/17562848221127238 36187365 PMC9520184

[91] Pöttgen J, Moss-Morris R, Wendebourg JM, Feddersen L, Lau S, Köpke S, et al. Randomised controlled trial of a self-guided online fatigue intervention in multiple sclerosis. J Neurol Neurosurg Psychiatry. 2018;89(9):970–6. doi: 10.1136/jnnp-2017-317463 29549193

[92] Rini C, Porter LS, Somers TJ, McKee DC, DeVellis RF, Smith M, et al. Automated Internet-based pain coping skills training to manage osteoarthritis pain: a randomized controlled trial. Pain. 2015;156(5):837–48. doi: 10.1097/j.pain.0000000000000121 25734997 PMC4402249

[93] Rocamora González C, Rodríguez Vega B, Torrijos Zarcero M, Mediavilla R, Bouzó Molina N, Plaza Fernández R, et al. Mindfulness based intervention through mobile app for colorectal cancer people awaiting surgery: A randomized clinical trial. Cir Esp (Engl Ed). 2022;100(12):747–54. doi: 10.1016/j.cireng.2022.08.008 36064177

[94] Schröder J, Brückner K, Fischer A, Lindenau M, Köther U, Vettorazzi E, et al. Efficacy of a psychological online intervention for depression in people with epilepsy: a randomized controlled trial. Epilepsia. 2014;55(12):2069–76. doi: 10.1111/epi.12833 25410633

[95] Schulz SM, Ritter O, Zniva R, Nordbeck P, Wacker C, Jack M, et al. Efficacy of a web-based intervention for improving psychosocial well-being in patients with implantable cardioverter-defibrillators: the randomized controlled ICD-FORUM trial. Eur Heart J. 2020;41(11):1203–11. doi: 10.1093/eurheartj/ehz134 30957867 PMC9597328

[96] van Beugen S, Ferwerda M, Spillekom-van Koulil S, Smit JV, Zeeuwen-Franssen ME, Kroft EB, et al. Tailored Therapist-Guided Internet-Based Cognitive Behavioral Treatment for Psoriasis: A Randomized Controlled Trial. Psychother Psychosom. 2016;85(5):297–307. doi: 10.1159/000447267 27508937

[97] van Luenen S, Garnefski N, Spinhoven P, Kraaij V. Guided internet-based intervention for people with HIV and depressive symptoms: a randomised controlled trial in the Netherlands. Lancet HIV. 2018;5(9):e488–e97. doi: 10.1016/S2352-3018(18)30133-4 30135045

[98] Young MD, Drew RJ, Kay-Lambkin F, Collins CE, Callister R, Kelly BJ, et al. Impact of a self-guided, eHealth program targeting weight loss and depression in men: A randomized trial. Journal of Consulting and Clinical Psychology. 2021;89(8):682–94. doi: 10.1037/ccp0000671 34472895

[99] Younge JO, Wery MF, Gotink RA, Utens EM, Michels M, Rizopoulos D, et al. Web-Based Mindfulness Intervention in Heart Disease: A Randomized Controlled Trial. PLoS One. 2015;10(12):e0143843. doi: 10.1371/journal.pone.0143843 26641099 PMC4671576

[100] Petkovic J, Trawin J, Dewidar O, Yoganathan M, Tugwell P, Welch V. Sex/gender reporting and analysis in Campbell and Cochrane systematic reviews: a cross-sectional methods study. Systematic Reviews. 2018;7(1):113. doi: 10.1186/s13643-018-0778-6 30068380 PMC6090880

[101] Doull M, Runnels VE, Tudiver S, Boscoe M. Appraising the evidence: applying sex- and gender-based analysis (SGBA) to Cochrane systematic reviews on cardiovascular diseases. J Womens Health (Larchmt). 2010;19(5):997–1003. doi: 10.1089/jwh.2009.1626 20384450

[102] Disparities NIoMaH. Diversity and Inclusion in Clinical Trials. In: Services Udoh, editor. 2023.

[103] Christiansen DM, McCarthy MM, Seeman MV. Editorial: Understanding the influences of sex and gender differences in mental disorders. Frontiers in Psychiatry. 2022;13. doi: 10.3389/fpsyt.2022.984195 36003972 PMC9393724

[104] Rosenwohl-Mack A, Tamar-Mattis S, Baratz AB, Dalke KB, Ittelson A, Zieselman K, et al. A national study on the physical and mental health of intersex adults in the U.S. PLoS One. 2020;15(10):e0240088. doi: 10.1371/journal.pone.0240088 33035248 PMC7546494

[105] Valentine SE, Shipherd JC. A systematic review of social stress and mental health among transgender and gender non-conforming people in the United States. Clin Psychol Rev. 2018;66:24–38. doi: 10.1016/j.cpr.2018.03.003 29627104 PMC6663089

[106] Reisner SL, Poteat T, Keatley J, Cabral M, Mothopeng T, Dunham E, et al. Global health burden and needs of transgender populations: a review. Lancet. 2016;388(10042):412–36. doi: 10.1016/S0140-6736(16)00684-X 27323919 PMC7035595

[107] Kundakovic M, Rocks D. Sex hormone fluctuation and increased female risk for depression and anxiety disorders: From clinical evidence to molecular mechanisms. Front Neuroendocrinol. 2022;66:101010. doi: 10.1016/j.yfrne.2022.101010 35716803 PMC9715398

[108] Cooper K, Russell A, Mandy W, Butler C. The phenomenology of gender dysphoria in adults: A systematic review and meta-synthesis. Clin Psychol Rev. 2020;80:101875. doi: 10.1016/j.cpr.2020.101875 32629301 PMC7441311

[109] Michos ED, Van Spall HGC. Increasing representation and diversity in cardiovascular clinical trial populations. Nature Reviews Cardiology. 2021;18(8):537–8. doi: 10.1038/s41569-021-00583-8 34108677

[110] Grace D. Intersectionality-informed Mixed Method Research: A Primer. The Institute for Intersectionality Research & Policy. 2014:1–22.

[111] Sharman Z, Johnson J. Towards the inclusion of gender and sex in health research and funding: an institutional perspective. Soc Sci Med. 2012;74(11):1812–6. doi: 10.1016/j.socscimed.2011.08.039 22000763

[112] Duchesne A, Tannenbaum C, Einstein G. Funding agency mechanisms to increase sex and gender analysis. Lancet. 2017;389(10070):699. doi: 10.1016/S0140-6736(17)30343-4 28229876

[113] Heidari S, Babor TF, De Castro P, Tort S, Curno M. Sex and Gender Equity in Research: rationale for the SAGER guidelines and recommended use. Res Integr Peer Rev. 2016;1:2. doi: 10.1186/s41073-016-0007-6 29451543 PMC5793986

[114] Schiebinger L, Leopold SS, Miller VM. Editorial policies for sex and gender analysis. Lancet. 2016;388(10062):2841–2. doi: 10.1016/S0140-6736(16)32392-3 27979394

[115] Merriman R, Galizia I, Tanaka S, Sheffel A, Buse K, Hawkes S. The gender and geography of publishing: a review of sex/gender reporting and author representation in leading general medical and global health journals. BMJ Glob Health. 2021;6(5). doi: 10.1136/bmjgh-2021-005672 33986001 PMC8118011

[116] Smith JA, Braunack-Mayer AJ, Wittert GA, Warin MJ. Qualities men value when communicating with general practitioners: implications for primary care settings. Med J Aust. 2008;189(11–12):618–21. doi: 10.5694/j.1326-5377.2008.tb02214.x 19061448

[117] Koch AR, Craemer KA, Garland CE, Fox WB, Jones CT, Qualls AC, et al. Federally Funded Randomized Controlled Trials Increase Analysis and Reporting of Study Outcomes by Sex, Race, and Ethnicity. J Womens Health (Larchmt). 2024;33(1):14–9. doi: 10.1089/jwh.2023.0307 37930690

[118] Hammack PL, Wignall L. Sexual and gender diversity in the twenty-first century. Current Opinion in Psychology. 2023;52:101616. doi: 10.1016/j.copsyc.2023.101616 37418831

[119] Phillips SP, Hamberg K. Doubly blind: a systematic review of gender in randomised controlled trials. Glob Health Action. 2016;9:29597. doi: 10.3402/gha.v9.29597 27087576 PMC4834361

