# Supplementary material for: Sex and gender reporting in RCTs of internet and mobile-based interventions for depression and anxiety in chronic conditions: A secondary analysis of a systematic review
Source: PLOS Ment Health. 2024 Jul 25;1(2):e0000048. doi: 10.1371/journal.pmen.0000048 (PMC12798592; doi:10.1371/journal.pmen.0000048)
Supplement: S1 Appendix — (DOCX) [file pmen.0000048.s001.docx]

**S1 Appendix: Full Search Strategy**

| **Database** | **Search String** |
| --- | --- |
| **MEDLINE** | 1. Exp mind-body therapies/ or exp breathing exercises/ or exp meditation/ or ("meditat*" or "wellness intervention" or "wellness therapy" or "wellness program" or mindful* or relaxation or "relaxation therapy" or yoga or "tai ji" or qigong or "breath* exercises" or mind-body or body-mind or "tai chi" or taichi or qigong or breath* or "guided imagery" or "acceptance commitment therapy" or "cognitive behavioural therapy" or "cognitive behavioral therapy" or vitalism or "meditative practice" or samu or "positive psycholog*" or "intentionally focused awareness" or simonton or visuali?ation or imagery or sophrology or "muscle contract*" or "hold relax" or "alternate nostril" or "abdominal breath" or neidan or "nei dan" or "unilateral nostril" or "forced nostril" or "deep breath*")   =603839     1. Exp Telemedicine/ or Exp Remote Consultation/ or (cyberthera* or telecare or telecollaborat* or teleconsult* or teleconference* or teleeducat* or telediagnos* or telehealth or teleguide* or telediagnos* or telelearn* or telemed* or telementor* or telemonitor* or teleneurol* or teleopth* or telepediatric* or telepresence* or telerehab* or telerobotic* or telescreen* or teletherap* or teletransmi* or mhealth or "m heath" or ehealth* or "e health" or website* or ((cyber or digital or remote* or distance* or tele) adj2 (medicine or care or collaborat* or consult* or conference* or educat* or diagnos* or health or guide* or diagnos* or learn* or med* or mentor* or monitor* or presence* or screen* or therap* or transmi*)) or ((cyber or digital or distance* or tele or remote* or sms or phone* or internet or "web based" or telephone* or texting or "mobile app*" or Instagram or Snapchat or Facetime or GMeet* or hangout* or Skype or Zoom or Web-ex or WebEx or Bluejeans or Facebook or e-mail* or email* or "e chat" or echat or "social media" or "text message*" or "answering machine*" or "voice mail*" or "video conferenc*" or "video link*" or "video chat*") adj3 (consult* or support* or diagnos* or "follow-up*" or "health" or doctor* or "primary care" or clinic or clinics or clinician* or nurs* or psycholog* or therap* or intervention* or delivery)))   =175839     1. exp mental disorders/ OR (depress* OR psych* OR behav* OR neuro* OR cognitive* OR anxiety OR attention* OR mental* health* OR stress).mp. OR stress, psychological.sh. OR (wellness OR qol OR "quality of life" OR SF36 OR SF-36)   =7459625     1. Exp Child/ or "Congenital, Hereditary, and Neonatal Diseases and Abnormalities"/ or exp infant/ or adolescent/ or exp pediatrics/ or child, abandoned/ or exp child, exceptional/ or child, orphaned/ or child, unwanted/ or minors/ or (pediatric* or paediatric* or child* or newborn* or congenital* or infan* or baby or babies or neonat* or pre-term or preterm* or premature birth* or NICU or preschool* or pre-school* or kindergarten* or kindergarden* or elementary school* or nursery school* or (day care* not adult*) or schoolchild* or toddler* or boy or boys or girl* or middle school* or pubescen* or juvenile* or teen* or youth* or high school* or adolesc* or pre-pubesc* or prepubesc*).mp. or (child* or adolesc* or pediat* or paediat*). jn   =5002496     1. Exp adult/ or adult*   =8559586     1. 1 & 2 & 3 = 5145      1. 6 NOT Reviews NOT (pediatrics NOT adults) = 4020 |
| **CINAHL** | 1. (MH "Yoga+") or (MH "Tai Chi") or (MH "Meditation") or (MH "Breathing Exercises+") or (MH "Qigong") or (MH "Mindfulness+") or  ("meditat*" or "wellness intervention" or "wellness therapy" or "wellness program" or mindful* or relaxation or "relaxation therapy" or yoga or "tai ji" or qigong or "breath* exercises" or mind-body or body-mind or "tai chi" or taichi or qigong or breath* or "guided imagery" or "acceptance commitment therapy" or "cognitive behavioural therapy" or "cognitive behavioral therapy" or vitalism or "meditative practice" or samu or "positive psycholog*" or "intentionally focused awareness" or simonton or visuali?ation or imagery or sophrology or "muscle contract*" or "hold relax" or "alternate nostril" or "abdominal breath" or neidan or "nei dan" or "unilateral nostril" or "forced nostril" or "deep breath*")   =134,013     1. (MH "Telemedicine+") OR (MH "Remote Consultation") or (((cyberthera* or telecare or telecollaborat* or teleconsult* or teleconference* or teleeducat* or telediagnos* or telehealth or teleguide* or telediagnos* or telelearn* or telemed* or telementor* or telemonitor* or teleneurol* or teleopth* or telepediatric* or telepresence* or telerehab* or telerobotic* or telescreen* or teletherap* or teletransmi* or mhealth or "m heath" or ehealth* or "e health" or (cyber or digital or remote* or distance* or tele)) N2 (medicine or care or collaborat* or consult* or conference* or educat* or diagnos* or health or guide* or diagnos* or learn* or med* or mentor* or monitor* or presence* or screen* or therap* or transmi*)) or ((cyber or digital or distance* or tele or remote* or sms or phone* or internet or "web based" or telephone* or texting or "mobile app*" or Instagram or Snapchat or Facetime or GMeet* or hangout* or Skype or Zoom or Web-ex or WebEx or Bluejeans or Facebook or e-mail* or email* or "e chat" or echat or "social media" or "text message*" or "answering machine*" or "voice mail*" or "video conferenc*" or "video link*" or "video chat*") N3 (consult* or support* or diagnos* or "follow-up*" or "health" or doctor* or "primary care" or clinic or clinics or clinician* or nurs* or psycholog* or therap* or intervention* or delivery)))   =68,817     1. (MH "mental disorders+") OR (depress* OR psych* OR behav* OR neuro* OR cognitive* OR anxiety OR attention* OR mental* health* OR stress).mp. OR stress, psychological.sh. OR (wellness OR qol OR "quality of life" OR SF36 OR SF-36)   =864,093     1. (pediatric* or paediatric* or child* or newborn* or congenital* or infan* or baby or babies or neonat* or “pre-term” or preterm or “premature birth*” or NICU or preschool* or “pre-school*” or kindergarten* or “elementary school*” or “nursery school*” or schoolchild* or toddler* or boy or boys or girl* or “middle school*” or pubescen* or juvenile* or teen* or youth* or “high school*” or adolesc*or prepubesc* or “pre-pubesc*” or (MH "Child+") OR (MH "Adolescence+") OR (MH "Minors (Legal)") or "(MH "Child Abuse, Sexual") OR (MH "Child Behavior Disorders+") OR (MH "Child, Medically Fragile") OR (MH "Child Day Care") OR (MH "Child Behavior+") OR (MH "Child Mortality") OR (MH "Child Passenger Safety") OR (MH "Child Development Disorders, Pervasive+") OR (MH "Child Custody") OR (MH "Child Abuse+") OR (MH "Child Nutritional Physiology+") OR (MH "Child Behavior Checklist") ) OR SO ( child* or pediatric* or paediatric* or adolescent* )   =1,531,799     1. (MH "adult+") or adult*   =2,184,798     1. 1 AND 2 AND 3   =941     1. 6 NOT (5 NOT 4)   =855 |
| **EMBASE** | 1. exp mindfulness/ or exp meditation/ or exp breathing exercise/ or exp yoga/ or exp tai chi/ or exp relaxation training/ or ("meditat*" or "wellness intervention" or "wellness therapy" or "wellness program" or mindful* or relaxation or "relaxation therapy" or yoga or "tai ji" or qigong or "breath* exercises" or mind-body or body-mind or "tai chi" or taichi or qigong or breath* or "guided imagery" or "acceptance commitment therapy" or "cognitive behavioural therapy" or "cognitive behavioral therapy" or vitalism or "meditative practice" or samu or "positive psycholog*" or "intentionally focused awareness" or simonton or visuali?ation or imagery or sophrology or "muscle contract*" or "hold relax" or "alternate nostril" or "abdominal breath" or neidan or "nei dan" or "unilateral nostril" or "forced nostril" or "deep breath*")   =909454     1. exp telemedicine/ or exp teleconsultation/ or (((cyberthera* or telecare or telecollaborat* or teleconsult* or teleconference* or teleeducat* or telediagnos* or telehealth or teleguide* or telediagnos* or telelearn* or telemed* or telementor* or telemonitor* or teleneurol* or teleopth* or telepediatric* or telepresence* or telerehab* or telerobotic* or telescreen* or teletherap* or teletransmi* or mhealth or "m heath" or ehealth* or "e health" or (cyber or digital or remote* or distance* or tele)) adj2 (care or collaborat* or consult* or conference* or educat* or diagnos* or health or guide* or diagnos* or learn* or med* or mentor* or monitor* or presence* or screen* or therap* or transmi*)) or ((cyber or digital or distance* or tele or remote* or sms or phone* or internet or "web based" or telephone* or texting or "mobile app*" or Instagram or Snapchat or Facetime or GMeet* or hangout* or Skype or Zoom or Web-ex or WebEx or Bluejeans or Facebook or e-mail* or email* or "e chat" or echat or "social media" or "text message*" or "answering machine*" or "voice mail*" or "video conferenc*" or "video link*" or "video chat*") adj3 (consult* or support* or diagnos* or follow-up* or health or doctor* or "primary care" or clinic or clinics or clinician* or nurs* or psycholog* or therap* or intervention* or delivery)))   =162959     1. exp mental disorders/ OR (depress* OR psych* OR behav* OR neuro* OR cognitive* OR anxiety OR attention* OR mental* health* OR stress).mp. OR stress, psychological.sh. OR (wellness OR qol OR "quality of life" OR SF36 OR SF-36) 2. 1 AND 2 AND 3 = 5725 3. juvenile/ or exp adolescent/ or exp child/ or exp postnatal development/ or (pediatric* or paediatric* or child* or newborn* or congenital* or infan* or baby or babies or neonat* or pre term or preterm* or premature birth or NICU or preschool* or pre school* or kindergarten* or elementary school* or nursery school* or schoolchild* or toddler* or boy or boys or girl* or middle school* or pubescen* or juvenile* or teen* or youth* or high school* or adolesc* or prepubesc* or pre pubesc*).mp. or (child* or adolesc* or pediat* or paediat*).jn.   =14518   1. Exp adult/ or adult* 2. 4 NOT (5 NOT 6) = 4964 |
| **PSYCInfo** | 1. exp mind body therapy/ or exp meditation/ or exp yoga/ or exp relaxation therapy/ or exp mindfulness/ or ("meditat*" or "wellness intervention" or "wellness therapy" or "wellness program" or mindful* or relaxation or "relaxation therapy" or yoga or "tai ji" or qigong or "breath* exercises" or mind-body or body-mind or "tai chi" or taichi or qigong or breath* or "guided imagery" or "acceptance commitment therapy" or "cognitive behavioural therapy" or "cognitive behavioral therapy" or vitalism or "meditative practice" or samu or "positive psycholog*" or "intentionally focused awareness" or simonton or visuali?ation or imagery or sophrology or "muscle contract*" or "hold relax" or "alternate nostril" or "abdominal breath" or neidan or "nei dan" or "unilateral nostril" or "forced nostril" or "deep breath*")   =137688     1. Exp Telemedicine/ or exp Teleconsultation/ or (cyberthera* or telecare or telecollaborat* or teleconsult* or teleconference* or teleeducat* or telediagnos* or telehealth or teleguide* or telediagnos* or telelearn* or telemed* or telementor* or telemonitor* or teleneurol* or teleopth* or telepediatric* or telepresence* or telerehab* or telerobotic* or telescreen* or teletherap* or teletransmi* or mhealth or "m heath" or ehealth* or "e health" or website* or ((cyber or digital or remote* or distance* or tele) adj2 (medicine or care or collaborat* or consult* or conference* or educat* or diagnos* or health or guide* or diagnos* or learn* or med* or mentor* or monitor* or presence* or screen* or therap* or transmi*)) or ((cyber or digital or distance* or tele or remote* or sms or phone* or internet or "web based" or telephone* or texting or "mobile app*" or Instagram or Snapchat or Facetime or GMeet* or hangout* or Skype or Zoom or Web-ex or WebEx or Bluejeans or Facebook or e-mail* or email* or "e chat" or echat or "social media" or "text message*" or "answering machine*" or "voice mail*" or "video conferenc*" or "video link*" or "video chat*") adj3 (consult* or support* or diagnos* or "follow-up*" or "health" or doctor* or "primary care" or clinic or clinics or clinician* or nurs* or psycholog* or therap* or intervention* or delivery)))   =65180     1. exp mental disorders/ OR (depress*  OR psych* OR behav* OR neuro* OR cognitive* OR anxiety OR attention* OR mental* health* OR stress).mp. OR stress, psychological.sh. OR (wellness OR qol OR "quality of life" OR SF36 OR SF-36)   =3682733     1. adolescent development/ or childhood development/  or pediatrics/ or exp Congenital Disorders/ or child characteristics/ or child abuse/ or exp child welfare/ or chronically ill children/  or child neglect/ or child psychiatry/ or child psychopathology/ or exp child care/  or (pediatric* or paediatric* or child* or newborn* or congenital* or infan* or baby or babies or neonat* or pre term or preterm* or premature birth or NICU or preschool* or pre school* or kindergarten* or elementary school* or nursery school* or schoolchild* or toddler* or boy or boys or girl* or middle school* or pubescen* or juvenile* or teen* or youth* or high school* or adolesc* or prepubesc* or pre pubesc*).mp. or (child* or adolesc* or pediat* or paediat*).jn.   =1413468     1. Exp adult attitudes/ or exp adult development/ or exp adult education/ or exp adult learning/ adult*   =817743     1. 1 AND 2 AND 3 = 3048 2. 6 NOT (4 NOT 5) = 2605 |
| **Cochrane** | 1. [mh "mind-body therapies"] or [mh "breathing exercises"] or [mh meditation] or ("meditat*" or "wellness intervention" or "wellness therapy" or "wellness program" or mindful* or relaxation or "relaxation therapy" or yoga or "tai ji" or qigong or "breath* exercises" or mind-body or body-mind or "tai chi" or taichi or qigong or breath* or "guided imagery" or "acceptance commitment therapy" or "cognitive behavioural therapy" or "cognitive behavioral therapy" or vitalism or "meditative practice" or samu or "positive psycholog*" or "intentionally focused awareness" or simonton or visuali?ation or imagery or sophrology or "muscle contract*" or "hold relax" or "alternate nostril" or "abdominal breath" or neidan or "nei dan" or "unilateral nostril" or "forced nostril" or "deep breath*"):ti,ab,kw = 85146     1. [mh Telemedicine] or [mh “Remote Consultation”] or (cyberthera* or telecare or telecollaborat* or teleconsult* or teleconference* or teleeducat* or telediagnos* or telehealth or teleguide* or telediagnos* or telelearn* or telemed* or telementor* or telemonitor* or teleneurol* or teleopth* or telepediatric* or telepresence* or telerehab* or telerobotic* or telescreen* or teletherap* or teletransmi* or mhealth or "m heath" or ehealth* or "e health" or website* or ((cyber or digital or remote* or distance* or tele) NEAR/2 (medicine or care or collaborat* or consult* or conference* or educat* or diagnos* or health or guide* or diagnos* or learn* or med* or mentor* or monitor* or presence* or screen* or therap* or transmi*)) or ((cyber or digital or distance* or tele or remote* or sms or phone* or internet or "web based" or telephone* or texting or "mobile app*" or Instagram or Snapchat or Facetime or GMeet* or hangout* or Skype or Zoom or Web-ex or WebEx or Bluejeans or Facebook or e-mail* or email* or "e chat" or echat or "social media" or "text message*" or "answering machine*" or "voice mail*" or "video conferenc*" or "video link*" or "video chat*") NEAR/3 (consult* or support* or diagnos* or "follow-up*" or "health" or doctor* or "primary care" or clinic or clinics or clinician* or nurs* or psycholog* or therap* or intervention* or delivery))):ti,ab,kw   = 39666     1. [mh "mental disorders"] OR (depress*  OR psych* OR behav* OR neuro* OR cognitive* OR anxiety OR attention* OR mental* health* OR stress) OR stress, psychological.sh. OR (wellness OR qol OR "quality of life" OR SF36 OR SF-36):ti,ab,kw   = 618 654     1. [mh "Child"] or ([mh "Congenital, Hereditary and Neonatal Diseases and Abnormalities"]) or [mh "infant"] or [mh ^"adolescent"] or [mh "pediatrics"] or [mh ^"child, abandoned"] or [mh "child, exceptional"] or [mh ^"child, orphaned"] or [mh ^"child, unwanted"] or [mh ^"minor"] or (pediatric* or paediatric* or child* or newborn* or congenital* or infan* or baby or babies or neonat* or pre-term or preterm* or (premature NEXT birth) or NICU or preschool* or (pre NEXT school*) or kindergarten* or kindergarden* or (elementary NEXT school*) or (nursery NEXT school*) or ((day NEXT care*) not adult*) or schoolchild* or toddler* or boy or boys or girl* or (middle NEXT school*) or pubescen* or juvenile* or teen* or youth* or (high NEXT school*) or adolesc* or (pre NEXT pubesc*) or prepubesc*):ti,ab,kw or (child* or adolesc* or pediat* or paediat*):so   = 344 420     1. [mh adult] or adult*:ti,ab,kw   = 860 684     1. #1 AND #2 AND #3   = 4367     1. #6 NOT (#4 NOT #5)   = 3918     1. Select only Cochrane trials = 3826 |
| **SCOPUS** | ( TITLE-ABS-KEY ( "mind-body therapies" OR "breathing exercises" OR meditation OR meditat* OR "wellness intervention" OR "wellness therapy" OR "wellness program" OR mindful* OR relaxation OR "relaxation therapy" OR yoga OR "tai ji" OR qigong OR "breath* exercises" OR "mind-body" OR "body-mind" OR "tai chi" OR taichi OR qigong OR breath* OR "guided imagery" OR "acceptance commitment therapy" OR "cognitive behavioural therapy" OR "cognitive behavioral therapy" OR vitalism OR "meditative practice" OR samu OR "positive psycholog*" OR "intentionally focused awareness" OR simonton OR visuali?ation OR imagery OR sophrology OR "muscle contract*" OR "hold relax" OR "alternate nostril" OR "abdominal breath" OR neidan OR "nei dan" OR "unilateral nostril" OR "forced nostril" OR "deep breath*") AND TITLE-ABS-KEY ( telemedicine OR "remote consultation" OR cyberthera* OR telecare OR telecollaborat* OR teleconsult* OR teleconference* OR teleeducat* OR telediagnos* OR telehealth OR teleguide* OR telediagnos* OR telelearn* OR telemed* OR telementor* OR telemonitor* OR teleneurol* OR teleopth* OR telepediatric* OR telepresence* OR telerehab* OR telerobotic* OR telescreen* OR teletherap* OR teletransmi* OR mhealth OR "m heath" OR ehealth* OR "e health" OR website* OR ( ( cyber OR digital OR remote* OR distance* OR tele ) W/2 ( medicine OR care OR collaborat* OR consult* OR conference* OR educat* OR diagnos* OR health OR guide* OR diagnos* OR learn* OR med* OR mentor* OR monitor* OR presence* OR screen* OR therap* OR transmi* ) ) OR ( ( cyber OR digital OR distance* OR tele OR remote* OR sms OR phone* OR internet OR "web based" OR telephone* OR texting OR "mobile app*" OR instagram OR snapchat OR facetime OR gmeet* OR hangout* OR skype OR zoom OR web-ex OR webex OR bluejeans OR facebook OR e-mail* OR email* OR "e chat" OR echat OR "social media" OR "text message*" OR "answering machine*" OR "voice mail*" OR "video conferenc*" OR "video link*" OR "video chat*" ) W/3 ( consult* OR support* OR diagnos* OR "follow-up*" OR "health" OR doctor* OR "primary care" OR clinic OR clinics OR clinician* OR nurs* OR psycholog* OR therap* OR intervention* OR delivery ) ) ) ) AND TITLE-ABS-KEY ( mental OR disorders OR depress* OR psych* OR behav* OR neuro* OR cognitive* OR anxiety OR attention* OR "mental* health*" OR " psychological stress" OR wellness OR qol OR "quality of life" OR sf36 OR sf-36 )  =6743 |
