# Supplementary figures and images for: Sex and gender reporting in RCTs of internet and mobile-based interventions for depression and anxiety in chronic conditions: A secondary analysis of a systematic review
Source: PLOS Ment Health. 2024 Jul 25;1(2):e0000048. doi: 10.1371/journal.pmen.0000048 (PMC12798592; doi:10.1371/journal.pmen.0000048)

**S2 Appendix: Risk of Bias Assessment**

**
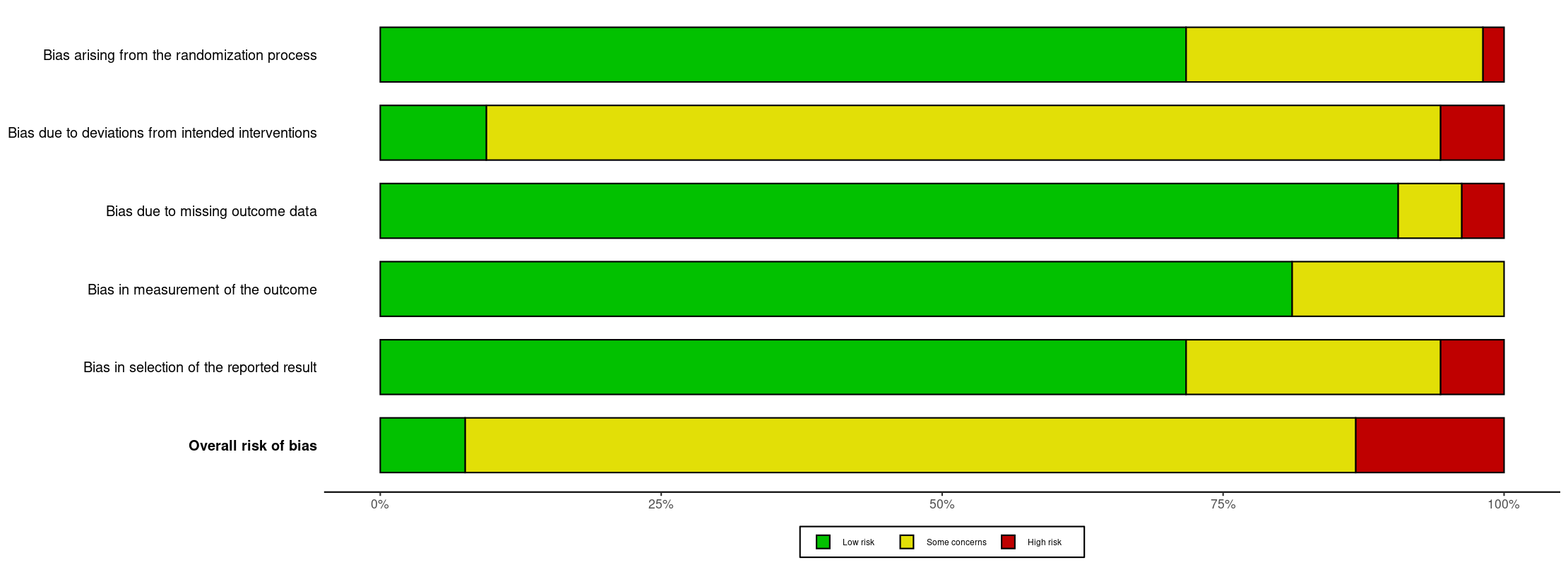
**

Supplement: S2 Appendix — (DOCX) [file pmen.0000048.s002.docx]
